# Pseudoepitheliomatous hyperplasia after diode laser oral surgery. An experimental study

**DOI:** 10.4317/medoral.20519

**Published:** 2015-06-27

**Authors:** Juan Seoane, Antonio González-Mosquera, José-Manuel García-Martín, Lucía García-Caballero, Juan-Manuel Seoane-Romero, Pablo Varela-Centelles

**Affiliations:** 1 Stomatology Department. School of Medicine and Dentistry. University of Santiago de Compostela. Spain. Entrerríos s/n. 15782. Santiago de Compostela. A Coruña. Spain; 2 Department of Surgery and Medical-Surgical Specialties. School of Medicine and Health Sciences. University of Oviedo. Spain. Campus del Cristo s/n. 22006 Oviedo. Spain; 3 EOXI Lugo, Cervo e Monforte de Lemos. Galician Health Service. Pza. Ferrol 11. 27001 Lugo. Spain

## Abstract

**Background:**

To examine the process of epithelial reparation in a surgical wound caused by diode laser.

**Material and Methods:**

An experimental study with 27 Sprage-Dawley rats was undertaken. The animals were randomly allocated to two experimental groups, whose individuals underwent glossectomy by means of a diode laser at different wattages, and a control group treated using a number 15 scalpel blade. The animals were slaughtered at the 2nd, 7th, and 14th day after glossectomy. The specimens were independently studied by two pathologists (blinded for the specimens’ group).

**Results:**

At the 7th day, re-epithelisation was slightly faster for the control group (conventional scalpel) (*p*=0.011). At the 14th day, complete re-epithelization was observed for all groups. The experimental groups displayed a pseudoepitheliomatous hyperplasia.

**Conclusions:**

It is concluded that, considering the limitations of this kind of experimental studies, early re-epithelisation occurs slightly faster when a conventional scalpel is used for incision, although re-epithelisation is completed in two weeks no matter the instrument used. In addition, pseudoepitheliomatous hyperplasia is a potential event after oral mucosa surgery with diode laser. Knowledge about this phenomenon (not previously described) may prevent diagnostic mistakes and inadequate treatment approaches, particularly when dealing with potentially malignant oral lesions.

**Key words:**Diode laser, animal model, oral biopsy, oral cancer, oral precancer, pseudoepitheliomatous hyperplasia.

## Introduction

Lasers with different wavelengths have been used for oral and maxillofacial surgery because of the features and affinities of each one (1), pursuing a minimally invasive approach, accuracy, negligible intraoperative hemorrhage, sterilization of the surgical wound, while causing infrequent postoperative morbidity (2). In this sense, diode laser (810 nm - 980 nm) has proved an efficient tool with a reliable performance, both in continuous (2-10 w) and pulsed mode (10-15 w), as well as on contact (focused) and non-contact (defocused) modes (3,4). Based upon these properties, recent clinical reports suggest diode laser might be the first choice for oral soft tissue surgery (5), particularly when dealing with biopsies for pre-malignant and malignant oral lesions (4,6).

Several clinical (7) and experimental studies (8,9) have revealed the presence of circulating tumour cells and a subsequent higher incidence of loco-regional metastases after incisional biopsies of oral squamous cell carcinomas (OSCC) undertaken with conventional scalpel. The number of cancer cells in circulation rests both on the cellular detachment from the primary tumour and on their accessibility to vessels during the removal of the tumour tissue sample (10). Yet, the use of a laser beam for biopsy may avoid seeding tumour cells, taking advantage of the laser capability for minimizing blood loss and sealing lymphatics and nerve endings (11). Despite these benefits, the use of lasers for biopsy purposes remains controversial, mostly due to the difficulty for the pathological assessment of the surgical margins (12).

Diode laser induces a thermal effect which causes epithelial damage and a hyalinised zone around the incision, sealing the vessels in the wound (13,14), that may be an advantage also when biopsing potentially malignant oral lesions (PMOL).

A number of cross-sectional studies have assessed the histological characteristics of both the incisions and surgical margins of oral lesions harvested with diode laser at different settings (4-6,13,14), but no longitudinal studies on this topic could be retrieved, despite the facts that histological findings change with time (15), and that re-epithelisation of the wound after incisional biopsies of PMOL or OSCC treatment is crucial. Thus, the aim of this study was to examine the process of epithelial reparation in a surgical wound caused by diode laser.

## Material and Methods

An experimental study with 27 adult Sprage-Dawley rats (weighted about 250 gr) was undertaken. The animals were randomly allocated to three groups: two experimental groups (n=9 in each), whose individuals underwent glossectomy by means of a diode laser at different wattages, and a control group (n=9) treated using a number 15 scalpel blade (B/Braun, Aesculap AG, Germany).

The diode laser (810nm) (Lasersmail, Biolase Technology, Inc.) was used in the pulsed mode at 5.1 watt, 25 Hz, 20 ms per pulse for one group, and in continuous mode at 6 watt for the other experimental group.

The calibration of the device (Lasersmail, Biolase Technology, Inc.) was performed using a power sensor (Ophir F100A-HE) and an OphirNova display. A 400 µm fibre -at a perpendicular incidence to the sensor- was used, placed 1-2 mm apart from it in order to minimize losses due to irregularities at the end of the fibre. Power density was calculated at the tip of the fibre (4480 W/cm2) for the continuous mode, and the energy density (fluency) for the pulsed mode (76.8 J/cm2).

The surgical technique (glossectomy) was undertaken by a single surgeon directing the laser beam perpendicular to the dorsum of the tongue while stabilising the specimen with a non-toothed Adson forceps applied to the tongue tip. The animals were slaughtered at the 2nd, 7th, and 14th day after glossectomy by an overdose of anesthesia, according to EU protocols. The study protocol was approved by the University of Santiago de Compostela Ethics Committee.

Following previously published protocols (16,17), the samples were immediately fixed in a 10% formal in-buffered saline solution for 24 hours. A single pathologist longitudinally cut all the specimens with a new disposable scalpel for each section. After routinely embedding in paraffin, sections were cut at 4 µm and stained with haematoxylin and eosin. The specimens were coded and independently studied by two pathologists (blinded for the specimens` group), until a consensus was reached for each case. The histological assessment included the whole lingual stump, aiming at evaluating the degree of epithelisation of the surgical wound. The slides were observed and photographed in a Provis AX70 microscope (Olympus Corp., Tokyo, Japan).

The Sinha scale (18,19) was used for assessing the degree of re-epithelisation: (0= re-epithelisation at the edge o the wound; 1= re-epithelisation covering less than half of the wound; 2= re-epithelisation covering more than half of the wound; 3=re-epithelisation covering the entire wound with irregular thickness; and 4= re-epithelisation covering the entire wound with regular thickness).

Statistical analysis was performed by means of a SPSS+ 15.0 statistical package (IBM, NY, USA). A non parametric test was used for comparing proportions (Chi square). The chosen significance level was 5%.

## Results

The diode laser caused limited or nonexistent intrasurgical bleeding.

Only one animal (allocated to the 6 watt group and scheduled for study at the 2nd day) was withdrawn from the study because of anaesthesia-related death in the first day of the follow-up period.

No signs of autolysis or phenomena related to inadequate tissue fixation could be observed.

**Figure 1:**
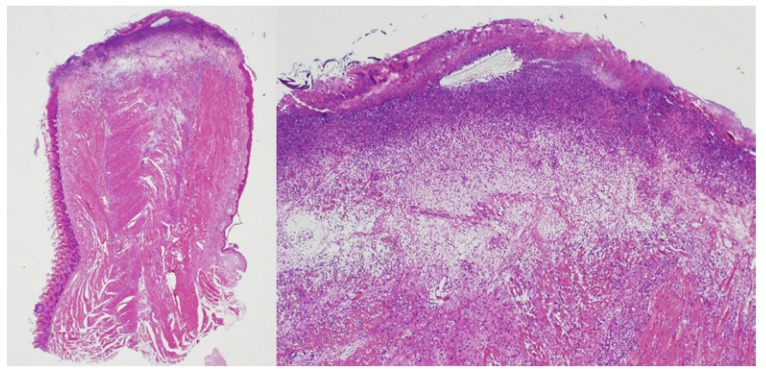
2nd day / diode laser in pulsed mode. At this initial phase of the wound healing, no re-epithelisation was found. The wound surface presented three layers over the muscle: a superficial eosinophilic layer composed by a fibrin matrix, a medium basophilic layer constituted by cell debris, and a deep clear oedematous layer (H&E, x1.25 left / x4 right).

The analyses performed at the second day after surgery showed absence of re-epithelisation (grade 0 Sinha’s scale) in all study groups (n=8;100%). At this moment, the surface of the wound was composed of fibrin strands covering debride, necrotic tissue with abundant, degenerated, poly nuclear neutrophils. Below the aforementioned sheet, a clear edematous layer with numerous neutrophils and mast cells lied over injured muscle fibres surrounded by inflammatory cells and occasional blood vessels and fibroblasts (Fig. 1).

**Figure 2:**
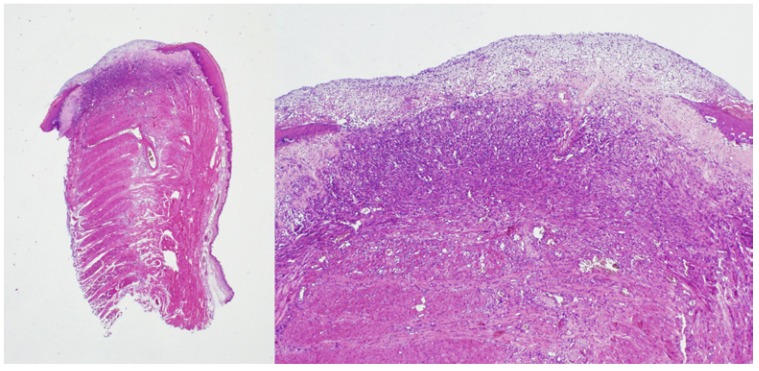
7th day / diode laser in pulsed mode. Seven days after surgery, re-epithelisation covered less than half of the wound surface, with an eosinophilic band of fibrosis below the newly formed epithelium. The wound surface showed a clear superficial layer, composed by fibrin and polynuclear neutrophils, that lies on a granulation tissue. (H&E, x1.25 left / x4 right).

At the 7th day, re-epithelisation was slightly faster for the control (conventional scalpel) vs experimental group (*p*=0.01). The whole control group (n=3;100%) scored grade 2 in Sinha’s scale -covering more than half of the wound- whereas the experimental groups (n=6) showed re-epithelisation covering less than half of the wound surface in 100% of the specimens, with fibrosis below the edges of the newly formed epithelium. In the centre of the wound, a layer of fibrin with poly nuclear neutrophils covered a granulation tissue with dilated blood vessels, proliferating fibroblasts, together with abundant lymphocytes and plasma cells (Fig. 2).

**Figure 3:**
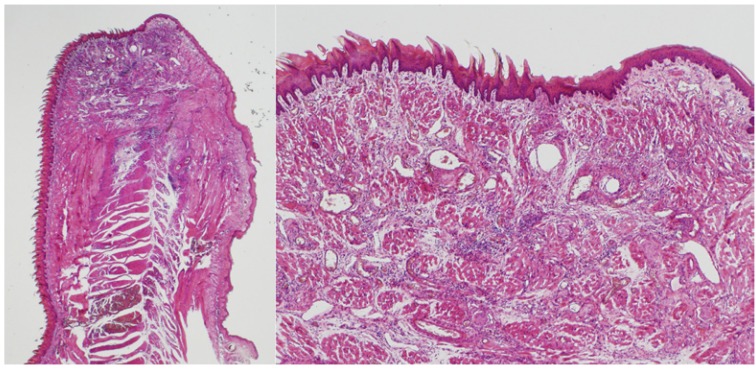
14th day / conventional scalpel. At the 14th day after surgery with conventional scalpel, the whole wound surface was covered by a normal epithelium. (H&E, x1.25 left / x4 right).

**Figure 4:**
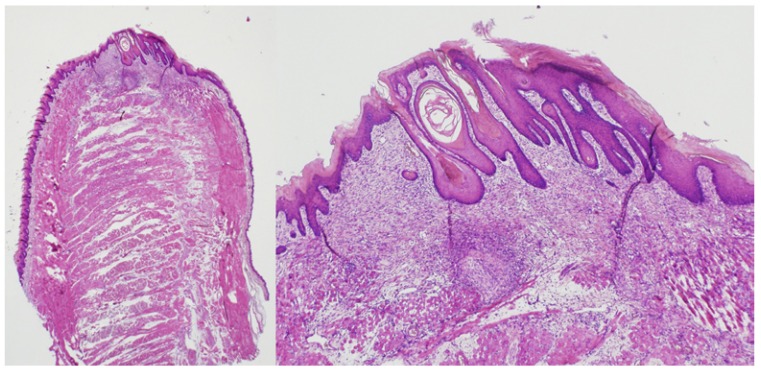
14th day / diode laser in pulsed mode. Complete wound re-epithelisation was also achieved after surgery with diode laser, but in these cases the epithelium showed a marked pseudoepitheliomatous hyperplasia. (H&E, x1.25 left / x4 right).

Fourteen days after surgery, a complete re-epithelisation of the wound could be observed for all groups, although significant differences were observed in terms of thickness of the epithelium between the control and experimental groups (*p*=0.01): in scalpel-treated specimens, a normal epithelium is formed (n=3; 100%) (Fig. 3), in contrast to the pseudoepitheliomatous hyperplasia found in the laser-treated samples (both in pulsed and continuous mode) (n=6; 100%). Light fibrosis, abundant inflammatory cells, and newly formed blood vessels and nerve endings were observed below the epithelium (Fig. 4).

## Discussion

A variety of animals and cadaverous materials have been proposed as experimental models for oral surgery techniques, and their validity for laser research has been formerly demonstrated (16,17). The thermal damage induced on oral soft tissues by diode lasers has been previously assessed in humans (6,20), ex-vivo porcine oral mucosa (13,14), and rats (15). This latter experimental model was selected for a longitudinal study (14 days) aimed at investigating the inflammatory response (15). In all the above mentioned reports, the surgical technique consisted in undertaking single or multiple superficial incisions (13-15).

Bearing in mind the aim of the present study was to assess the re-epithelisation of a surgical wound -resembling that produced after an incisional biopsy- and also that oral biopsies obtained by means of a diode laser require at least a diameter of 5 mm to be morphologically interpretable (6), we have chosen the protocol of glossectomy in rats (16,17). This surgical model has been previously used for assessing the impact of CO2 and Er,Cr:YSGG lasers on oral tissues (16,17).

Diode lasers at defocused mode (10-15 w/ continuous wave) permit vaporisation of leukoplastic and lichen planus oral lesions (5,21), but clinical studies have used 2 to 7 watt outputs for biopsing pre malignant and malignant oral lesions (4). More specifically, a diode laser of 810 nm, at 5.2 w, pulsed mode, using a 400 µm diameter fibre, in contact-type treatment mode, permits biopsy taking without significant complications (20). These findings seem to support the settings of the laser device used in our experimental model.

The diode laser’s thermal effect is widely known and may affect up to 754.2 µm (95%CI=551.0-957.4) of tissue at both sides of the wound (4,6,13,20). These effects include thermal cytological artifactual epithelial changes consisting in alteration of the cell structure and cytological atypias (4,6), which may resemble pseudo-dysplastic changes (presence of fusiform cells, hypechromatism, polymorphism and nuclear elongation, with loss of intercellular adherence). These findings have been previously described for CO2 and Er,Cr:YSGG lasers when used on oral mucosa (12,17). Misdiagnosis of these changes may well lead to a wrong therapeutic approach, as these criteria for dysplasia are particularly relevant for predicting the malignant potential of the lesion (22). In order to prevent this undesired circumstance, it has been suggested to enlarge the surgical incision (including an additional amount of healthy marginal tissue) at least 0.5 mm when dealing with suspected dysplastic or neoplastic lesions (6). However, wider tissue damage has also been reported from in vivo studies in humans when using a diode laser, which may support the idea of enlarging the amount of healthy tissue in the sample up to 3 mm (20). Despite re-epithelization of the surgical wound may condition lesion recurrence (23) and also that it is an essential event in the interpretation of specimens obtained either after incisional biopsies, or OSCC treatment (included in follow-up protocols) (24), we could not retrieve any study focused on epithelial repair after diode laser irradiation.

Our results, obtained under experimental conditions, unveiled the existence of pseudoepitheliomatous hyperplasias (PEH) in the diode laser groups. This finding severely compromises the suitability of this laser as an adjunctive surgical instrument for taking oral soft tissue biopsies (6), particularly when dealing with epithelial pre-neoplastic or neoplastic disorders.

PEH is a reactive epithelial proliferation whose main microscopic features include a prominent irregular epithelial hyperplasia, with epithelial barbs in a pseudo-invasive pattern towards the connective tissue. Due to these characteristics, PEH is also known as “pseudocarcinomatous hyperplasia” (25). Within the oral cavity, PEH has been described in association to granular cell tumours, necrotizing sialometaplasia, paracoccidioidomycosis, bisphosphonate-related osteonecrosis, oral sub mucous fibrosis, and pleomorphic adenoma of minor salivary glands. Its similarity to a OSCC may also lead to a wrong diagnosis (24,25). This diagnostic difficulty increases when dealing with small, superficial samples with a high inflammatory component and poorly oriented tissue sections (24). Besides, no consensus has been reached on an immunohistochemical panel to differentiate PEH from OSCC (24), so knowledge about the PEH phenomenon after diode laser irradiation is particularly relevant.

It may well have occurred that the scarcity of longitudinal studies and the use of superficial incisions as a surgical model (15) had prevented an earlier description of this finding. PEH has also been reported in burns and contaminated chronic wounds, where appears between the 10th and the 14th day after the injury (25).

Our experimental model has provided similar conditions to that of the actual clinical situation, with an adequate follow-up and a wide surgical surface exposed to bacterial contamination. On the other hand, our model also includes non-specialised lingual mucosa in the surgical wound because lingual squamous cell carcinomas and potentially malignant disorders usually arise in non-specialised mucosa at the lateral borders of the tongue. Furthermore, this reactive epithelial hyperplasia (PEH) in the oral mucosa seems to be originated from salivary glands ducts and, occasionally, in the mucous superficial epithelium. However, the fact that it has been more frequently found in mucous surfaces rich in salivary glands seems to suggest a more likely glandular origin (25).

It is concluded that, considering the limitations of this kind of experimental studies, early re-epithelisation occurs slightly faster when a conventional scalpel is used for incision, although re-epithelisation is completed in two weeks no matter the instrument used. In addition, pseudoepitheliomatous hyperplasia is a potential event after oral mucosa surgery with diode laser. Knowledge about this phenomenon (not previously described) may prevent diagnostic mistakes and inadequate treatment approaches, particularly when dealing with potentially malignant oral lesions.
